# Intelligent Fault Diagnosis of Hydraulic Pumps Based on Multi-Source Signal Fusion and Dual-Attention Convolutional Neural Networks

**DOI:** 10.3390/s25227018

**Published:** 2025-11-17

**Authors:** Wanlu Jiang, Xiaoyang Gu, Yonghui Zhao, Enyu Tang, Xu Jiang, Zixu Song, Linghui Zeng

**Affiliations:** 1 National-Local Joint Engineering Research Center for Advanced Manufacturing, Forming Technology and Equipment, Yanshan University, Qinhuangdao 066004, China; wljiang@ysu.edu.cn (W.J.); ysuzyh1996@163.com (Y.Z.); ysyytey@163.com (E.T.); ysujiangxu@163.com (X.J.); ysusongzixu@163.com (Z.S.); ysuzlh@163.com (L.Z.); 2 Hebei Provincial Key Laboratory of Heavy Machinery Fluid Power Transmission and Control, Yanshan University, Qinhuangdao 066004, China

**Keywords:** multi-source signal fusion, deep learning, dual-attention mechanism, convolutional neural networks, hydraulic pump, fault diagnosis

## Abstract

As a core component of hydraulic systems, hydraulic pumps generate vibration signals that contain abundant key features reflecting the operational state of internal machinery. However, most existing fault diagnosis methods rely solely on single-channel vibration data, neglecting the correlations and complementarities among multi-channel signals, which results in unstable and less accurate diagnostic outcomes. To address this limitation, this study proposes an intelligent fault diagnosis approach for hydraulic pumps based on multi-source signal fusion and a dual attention mechanism. First, vibration, pressure, and acoustic signals are transformed into time-frequency feature images, and an RGB image fusion strategy is applied to map the time-frequency representations of different signals into the individual channels of a color image. Subsequently, a convolutional neural network incorporating enhanced channel and spatial attention mechanisms is constructed to extract features from the fused images and perform classification. Experimental results demonstrate that the proposed method significantly improves fault diagnosis performance and outperforms other deep learning-based approaches, offering a novel strategy for intelligent hydraulic pump diagnostics with promising engineering applications.

## 1. Introduction

Hydraulic systems are essential core subsystems for power transmission and control in modern industrial equipment and have been widely applied in high-end fields such as aerospace, marine engineering, and precision manufacturing [1,2]. The hydraulic pump, as the key power source of these systems, operates under complex conditions including high temperature, high pressure, and heavy loads for extended periods, making its critical components prone to wear, cracking, and other faults. Faults not only compromise system stability and reliability but, in severe cases, may also lead to production interruptions or major safety incidents [3]. In practical engineering applications, hydraulic pumps are characterized by strong sealing, complex operating environments, and weak signal features that are easily masked by noise, all of which reduce the accuracy of traditional diagnostic models.

With the advancement of artificial intelligence, deep learning—owing to its powerful feature extraction and pattern recognition capabilities—has been widely adopted in the field of industrial intelligence, demonstrating particular advantages in fault diagnosis tasks. Traditional fault diagnosis methods, which rely on manually designed features and empirical rules, suffer from limitations such as poor generalization and weak robustness [4,5]. In contrast, deep learning-based intelligent fault diagnosis methods can automatically extract latent features from raw data, thereby avoiding information loss caused by human intervention. Consequently, deep learning-based fault diagnosis techniques are gradually becoming a research focus in this field and show promising potential for practical engineering applications. For example, Xu et al. [6] proposed an improved multi-scale convolutional neural network (CNN) incorporating a feature attention mechanism to address the poor diagnostic performance of traditional CNN models under complex operating conditions. Experimental results demonstrated that the method achieved promising performance on wind turbine bearing datasets. In [7], a rotating machinery fault diagnosis approach combining variational mode decomposition (VMD) with CNN was proposed. Furthermore, Wang et al. [8] proposed a fault diagnosis method for axial piston pumps based on deep belief networks (DBNs). By layer-wise stacking of restricted Boltzmann machines, the DBN can automatically learn fault features, achieving a classification accuracy of 97.40%, which validates the effectiveness of the approach.

As a representative deep learning model, convolutional neural networks (CNNs) excel in tasks such as speech recognition and image processing due to structural advantages including local receptive fields, weight sharing, and multiple convolutional kernels [9,10]. In fault diagnosis, transforming one-dimensional time-series signals into images not only enhances their representation in the feature space but also provides a more intuitive depiction of latent fault characteristics, thereby facilitating the extraction of deep structural information by models. Consequently, the integration of signal imaging with deep learning has emerged as a key research focus in the field of mechanical fault diagnosis. For example, Lian et al. [11] proposed a time-series image generation scheme that converts one-dimensional temporal signals into grayscale images, providing an intuitive representation of fault features and enhancing computational efficiency by optimizing the CNN architecture. This method demonstrated superior performance in fault diagnosis for motor bearings, self-priming centrifugal pumps, and hydraulic pumps. In [12], a method for rolling bearing fault diagnosis was proposed, combining neural networks with time-frequency domain analysis of bearing vibrations. In [13], raw time-domain signals were converted into two-dimensional grayscale images and subsequently input into a CNN for fault identification. In [14], Continuous Wavelet Transform (CWT) was employed for time-frequency analysis of vibration signals, and the resulting time-frequency maps were fed into a CNN for fault classification. Furthermore, Wang et al. [15] introduced a novel intelligent bearing fault diagnosis method based on symmetric point pattern representation and extrusion-excited convolutional neural networks. This approach achieved over 99% classification accuracy while demonstrating strong generalization capability and stability.

Although deep learning has achieved significant advances in mechanical fault diagnosis, most existing studies still rely on single-sensor inputs, limiting the ability to fully capture multi-dimensional information during equipment operation. In contrast, multi-sensor data fusion provides richer redundancy and complementary features, enabling a more comprehensive representation of equipment status and thereby enhancing the accuracy and robustness of fault diagnosis [16,17,18]. In this study, vibration, pressure, and acoustic signals of the hydraulic pump were simultaneously collected and transformed into time-frequency representations using the Short-Time Fourier Transform (STFT). The resulting images, after discretization and normalization, were mapped to the individual channels of RGB images to generate fused pseudo-color representations, which were then fed into a convolutional neural network for automatic identification and classification of the hydraulic pump’s fault states.

The remainder of this paper is organized as follows. Section 2 presents the theoretical foundations, including CNN and STFT. Section 3 provides a detailed description of the proposed hydraulic pump fault diagnosis method. Section 4 outlines the experimental setup and analyzes the results to validate the effectiveness of the proposed approach. Finally, Section 5 concludes the paper.

## 2. Basic Theory

### 2.1. Convolutional Neural Networks

**Figure 1:**
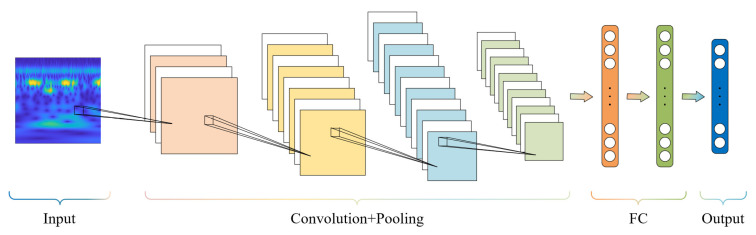
Convolutional Neural Network Architecture.

Convolutional neural networks are deep learning models widely used for image recognition and classification tasks. As shown in Figure 1, a typical CNN architecture comprises an input layer, convolutional layers, pooling layers, fully connected layers, and an output layer [19,20,21]. Compared with other deep learning models, CNNs offer three primary advantages: local connectivity, weight sharing, and pooling. These structural characteristics not only enhance feature extraction efficiency but also reduce the risk of overfitting, rendering CNNs a powerful tool for image processing and pattern recognition.

As the core computational unit in a CNN, the convolutional layer performs the majority of the network’s computations. Convolutional layers extract higher-level features by applying convolution operations on local regions of the input using a series of convolutional kernels [22]. The convolutional kernels slide across the input, allowing local receptive fields to cover the entire input and thereby capture spatial hierarchical features within the data. The output of the convolution operation undergoes a nonlinear transformation via an activation function, generating new feature maps that are passed to subsequent layers [19,23]. The convolution operation can be expressed by the following formula:

(1) $$x_{j}^{l}=f\left(\sum_{i\in M_{j}}x_{i}^{l-1}*k_{ij}^{l}+b_{j}^{l}\right)$$

In the formula, $x_{j}^{l}$ denotes the output of the l-th layer, $M_{j}$ represents the set of input feature maps connected to the j-th output feature map, and $x_{i}^{l-1}$ is the output of the $\left(l\;-\;1\right)$-th layer. $k_{ij}^{l}$ is the corresponding convolution kernel, and the convolution operation is denoted by $\left(*\right)$. $b_{j}^{l}$ represents the bias, while $f\left(\cdot \right)$ denotes the activation function, which enhances the CNN’s capability to model nonlinear relationships.

(2) $$f\left(x\right)=\max\left(0,x\right)$$

The pooling layer, also referred to as the downsampling layer, is typically positioned after the convolutional layers. Its primary function is to reduce the spatial dimensions of the input feature maps while preserving translation invariance, thereby improving the model’s convergence and generalization and mitigating the risk of overfitting. Pooling layers enable the extraction of more informative features from the outputs of the convolutional layers. The operation of the pooling layer can be expressed as:

(3) $$a_{j-s}^{l}=f\left(w_{j}^{l}\cdot \mathrm{down}\left(M_{j}^{l-1}\right)+b_{j}^{l}\right)$$

In the formula, $a_{j-s}^{l}$ denotes the activation output of the j−s-th channel in the l-th layer, s represents the channel offset, $w_{j}^{l}$ denotes the weight parameter, $b_{j}^{l}$ is the bias, and $M_{j}^{l-1}$ is the feature map of the j-th channel from the previous convolutional layer. $\mathrm{down}\left(\cdot \right)$ represents the pooling operation, typically either max pooling or average pooling.

In practical applications of CNNs, multiple convolutional and pooling layers are typically stacked to progressively extract features from images. As the network depth increases, the extracted representations become increasingly rich and abstract. After multiple alternating convolution and pooling operations, the feature maps from the previous layer are flattened and concatenated into a feature vector, which is then fed into the fully connected layer. In the fully connected layer, each neuron is connected to all neurons in the preceding layer, enabling the integration of discriminative features extracted by the convolutional and pooling layers to produce the final classification output. The mathematical expression is given as follows:

(4) $$y^{k}=f\left(W^{k}x^{k-1}+b^{k}\right)$$

In the formula, $y^{k}$ denotes the output of the *k*-th fully connected layer, $x^{k-1}$ is the flattened one-dimensional feature vector, $W^{k}$ represents the weight matrix, $b^{k}$ is the bias term, and $f\left(\cdot \right)$ denotes the activation function.

**Figure 2:**
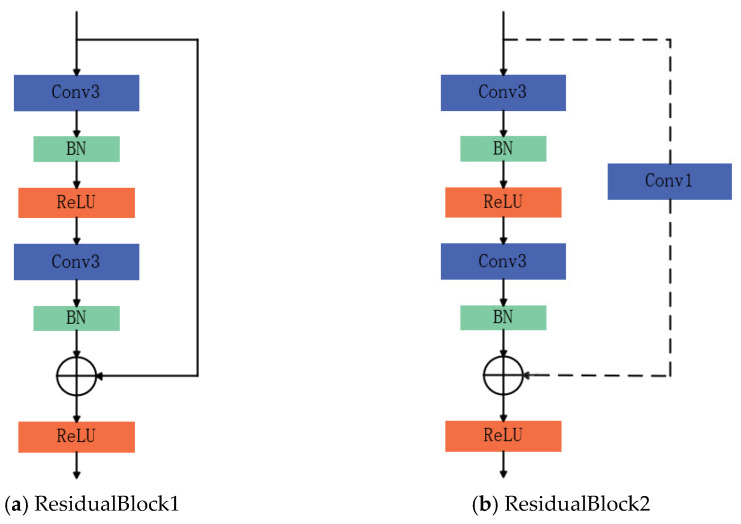
Two Typical Types of Residual Block Structures.

ResNet-18 is a relatively lightweight yet representative model within the residual network family, and its overall architecture primarily comprises two typical residual modules: ResidualBlock1 and ResidualBlock2 [24]. As shown in Figure 2a, ResidualBlock1 consists of two convolutional layers (Convs) responsible for feature extraction and transformation, with Batch Normalization (BN) applied after each convolutional layer to improve training stability and accelerate convergence. A ReLU activation function is applied at the end of the module to enhance nonlinear representation and facilitate feature integration.

As shown in Figure 2b, ResidualBlock2 further incorporates additional convolutional layers to enable more complex feature extraction and representation. Nonlinear integration and output are similarly achieved through the ReLU activation function. Both residual modules employ either identity mapping or projection mapping strategies to merge the input with the main branch output, thereby ensuring effective gradient propagation within the deep network.

### 2.2. Short-Time Fourier Transform

STFT is a widely used and easily implementable signal processing technique that converts one-dimensional time-series signals into two-dimensional time–frequency representations. This method frames the signal along the time axis, dividing it into multiple short segments and performing a Fourier transform on each segment to obtain local spectral information [25,26]. As the analysis window slides over time, the STFT depicts the signal’s frequency distribution at different time points. Due to its capability for localized analysis in both time and frequency, STFT is well suited for analyzing non-stationary signals whose spectra vary over time.

For a continuous non-stationary signal $x\left(t\right)$, its STFT is defined as

(5) $$\mathrm{STFT}\left(\tau ,\omega \right)=\int_{-\infty }^{\infty }x\left(t\right)w*(t-\tau )e^{-j\omega t}dt$$

Here, t denotes time, ω denotes angular frequency, j is the imaginary unit, and $w*\left(t\right)$ is the complex conjugate of the window function. The STFT essentially performs a Fourier transform on the signal $x\left(t\right)$ weighted by the window function at time τ.

In practical applications, continuous-time signals are typically discretized, and the discrete expression is given as follows

(6) $${\mathrm{STFT}}_{x}\left(n,k\right)=\sum_{m=-\infty }^{\infty }x\left(m\right)w\left(n-m\right)e^{-j\frac{2\pi }{N}km}$$

Here, *x*(*m*) denotes the discrete-time signal, *w*(*n* − *m*) represents the window function centered at *n*, *m* is the index of the signal’s time sampling points and also serves as the summation variable, *n* indicates the center position of the current window, *k* is the frequency index, and *N* is the number of Fourier transform points.

## 3. Research Methodology

With the advancement of sensor technology, both the number and types of sensors in hydraulic systems have been increasing. These sensors are commonly employed to monitor the real-time operating conditions of equipment, including vibration, pressure, sound, and temperature. By providing complementary information on the hydraulic pump’s operational state from multiple perspectives, they facilitate a comprehensive assessment of its health. Compared with single-signal diagnostic approaches, multi-source signal fusion effectively integrates these complementary data, thereby enhancing the accuracy of fault detection. Therefore, this study introduces a multi-source signal fusion strategy to achieve a more comprehensive characterization of hydraulic pump fault features by integrating complementary information from multiple sensors. The proposed methodology comprises three key components. First, a data-level fusion approach is developed to construct multi-source signal RGB images, enabling the visual integration of signals from different sources. Second, an enhanced ResNet-18 network is designed, incorporating a dual attention mechanism—both channel and spatial—to improve the network’s sensitivity to critical features. Finally, a complete fault diagnosis framework is established to facilitate the automatic recognition and classification of various hydraulic pump fault types.

### 3.1. RGB Image Fusion Method for Multi-Source Signals

According to the level of fusion, data fusion methods are generally categorized into three types: data-level fusion, feature-level fusion, and decision-level fusion [19,27]. Data-level fusion is the most primitive form, which directly combines raw data and typically requires time alignment and signal normalization. Feature-level fusion first extracts features from each sensor channel separately, and then integrates them through feature concatenation, weighted combination, or feature mapping transformations [27]. Decision-level fusion aggregates the diagnostic outputs from multiple sub-models using methods such as voting, weighted averaging, or Bayesian inference [17]. The major advantages and disadvantages of these three approaches are summarized in Table 1.

This study proposes a method for fusing the time–frequency features of signals into RGB images, which belongs to data-level fusion. Compared with the other two fusion approaches, data-level fusion can fully preserve the original information of each signal. Particularly in scenarios with large sample sizes or requiring rapid deployment, this method offers practical advantages such as simplicity of implementation, high efficiency, and strong adaptability, making it suitable for widespread application in multi-sensor equipment condition monitoring. Specifically, let the multi-source signals of the hydraulic pump be

(7) $$X=\left\{x_{1}\left(t\right),x_{2}\left(t\right),x_{3}\left(t\right)\right\}$$

here $x_{i}\left(t\right)$ denotes the signal from the i−th sensor (e.g., vibration, pressure, or acoustic signal).

First, a short-time Fourier transform is applied to each type of signal to obtain its time–frequency magnitude matrix:

(8) $$S_{i}\left(f,\tau \right)=\mathrm{STFT}\left(x_{i}\left(t\right)\right),i=1,2,3$$

here f denotes the frequency index, and τ denotes the time-window index.

Subsequently, the magnitude matrix is normalized to the range [0, 255] to obtain a grayscale image matrix:

(9) $$G_{i}\left(f,\tau \right)=255\cdot \frac{\left|Si\left(f,\tau \right)\right|-\min\left(\left|S_{i}\right|\right)}{\max\left(\left|S_{i}\right|\right)-\min\left(\left|S_{i}\right|\right)},i=1,2,3$$

Finally, the three grayscale matrices are assigned to the R, G, and B channels, respectively, to construct an RGB image that integrates the features of the three types of signals.

(10) $$I_{RGB}\left(f,\tau \right)=cat_{3}\left[G_{1}\left(f,\tau \right),G_{2}\left(f,\tau \right),G_{3}\left(f,\tau \right)\right]$$

Under different fault conditions, the sensor signals exhibit significant variations in frequency distribution and energy structure. These differences are clearly reflected in the color intensity and texture patterns of the RGB images, providing deep models with rich and distinctive input features for effective feature extraction. The pseudocode describing the RGB image generation process from multi-channel signals using STFT is presented in Algorithm 1.
**Algorithm 1.** RGB Image Generation Process from Multi-Channel Signals Using STFT**Step****Description****Input:**Multi-channel signals *x(t)*, *p(t)*, *s(t)* with sampling rate *f_s_*.**Output:**RGB fusion images *I_RGB_*_._**Step 1:**For each signal segment *x_i_(t)*, *p_i_(t)*, *s_i_(t)*:**Step 2:****Z-score Normalization** Standardize each segment to have zero mean and unit variance to ensure signals are on the same scale; applied to all channels**Step 3:****STFT Computation** Compute Short-Time Fourier Transform (STFT) for each segment and limit the frequency range to a specified upper bound.**Step 4:**Convert STFT magnitudes to decibels (dB) and normalize to the range [0,1].**Step 5:**Resize each channel image to a fixed size.**Step 6:**Combine the three channel images to form an RGB image.**Step 7:**Save the generated RGB image as a PNG file, naming according to class and index

The proposed fusion method demonstrates high versatility and adaptability. It is applicable not only to scenarios involving the fusion of heterogeneous sensor signals, such as the vibration, pressure, and sound signals considered in this study, but can also be extended to the fusion of homogeneous sensor signals. Where spatial constraints permit, vibration sensors can be installed along three orthogonal directions (x, y, z) at key locations of the equipment. The signals from each axis can be processed individually and mapped to the R, G, and B channels, thereby enabling a joint representation of multi-axis vibration signals.

To verify the feasibility and effectiveness of the proposed RGB image fusion method, three simulated signals were constructed for testing, with a sampling frequency of 1000 Hz. Each signal comprises an exponential decay term and a phase-modulated cosine function, expressed as follows:

(11) $$\left\{\begin{array}{c} s_{1}(t)=e^{-0.2t}\times \cos(300\pi t+\cos(40\pi t))\\ s_{2}(t)=e^{-0.3t}\times \cos(500\pi t+\cos(50\pi t))\\ s_{3}(t)=e^{-0.5t}\times \cos(700\pi t+\cos(60\pi t)) \end{array}\right.$$

Here, the exponential decay term $e^{-\alpha t}$ is used to simulate the energy decay observed in real signals over time. The primary oscillation term adopts a typical phase-modulated form, $\cos\left(\omega t+\cos(\omega_{m}t)\right)$, where the carrier frequencies are 150 Hz, 250 Hz, and 350 Hz, and the modulation frequencies are 20 Hz, 25 Hz, and 30 Hz, respectively.

**Figure 3:**
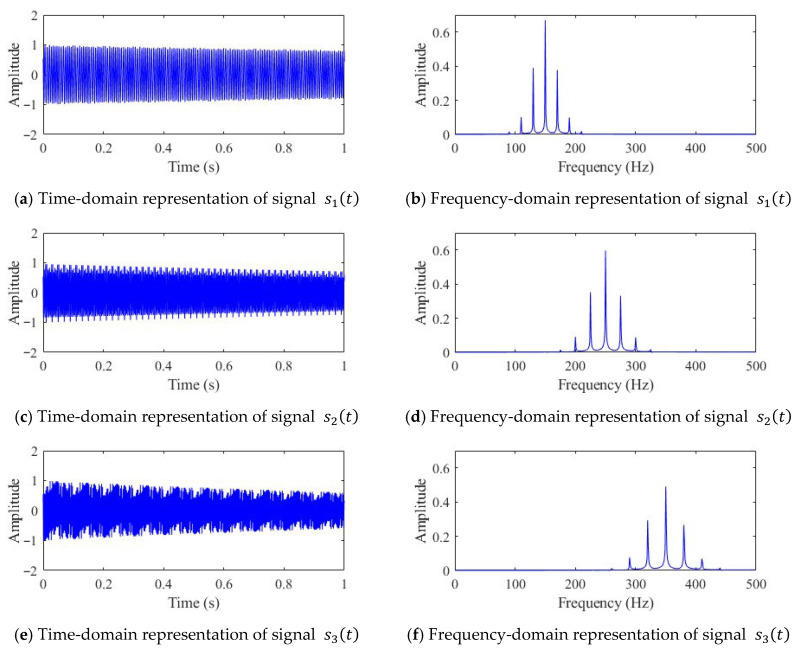
Time- and frequency-domain representations of the simulated signal.

The three sets of signals exhibit pronounced non-stationary characteristics and are widely used to simulate the instantaneous frequency variations commonly encountered in practical engineering. The exponential decay component reflects the gradual attenuation of signal energy over time, consistent with the characteristics of impact processes occurring during faults. Meanwhile, the phase modulation component, achieved by introducing a low-frequency cosine modulation into the carrier frequency, induces a slow variation in the signal frequency over time, closely approximating the dynamic response of equipment under real operating conditions. The time-domain waveforms and corresponding spectra of the simulated signals are shown in Figure 3.

**Figure 4:**
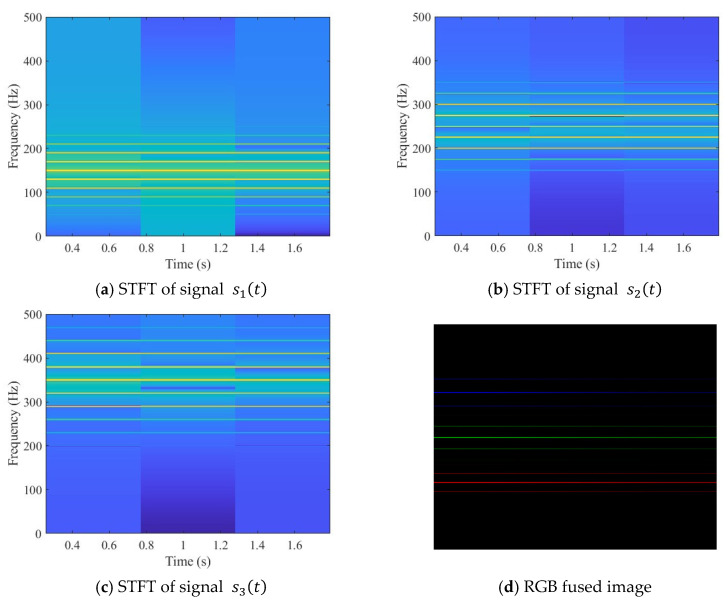
STFT time–frequency representation of the simulated signal and the RGB fused image.

Figure 4 presents the time-frequency representations and the RGB image fusion results of the three signals after STFT processing, with 2048 sampling points uniformly extracted for each simulated signal. From the STFT time-frequency maps, signal $s_{1}\left(t\right)$ exhibits multiple bright horizontal bands in the low-frequency region, indicating that its frequency components are predominantly concentrated in the low-frequency range. Signal $s_{2}\left(t\right)$ shows several continuous and prominent spectral lines in the mid-frequency region, consistent with its spectral characteristics. Signal $s_{3}\left(t\right)$ displays distinct bright bands in the high-frequency region, particularly around 350 Hz where multiple clear high-intensity spectral lines appear, demonstrating that its frequency components are primarily distributed in the high-frequency band.

In this study, signals $s_{1}\left(t\right)$, $s_{2}\left(t\right)$ and $s_{3}\left(t\right)$ are mapped to the red (R), green (G), and blue (B) channels of an RGB image, respectively, to construct a three-channel image that fuses information from all three signals. In the fused image, the lower red-striped region represents the low-frequency features of $s_{1}\left(t\right)$, the central green region corresponds to the mid-frequency features of $s_{2}\left(t\right)$, and the upper blue bright band reflects the high-frequency characteristics of $s_{3}\left(t\right)$. Variations in color saturation within the fused image indicate the amplitude of the STFT for the corresponding channel, the higher saturation corresponds to stronger signal energy in that frequency range.

Simulation results indicate that the constructed RGB fusion images exhibit color distributions that closely correspond to the frequency structures of the individual signals. This fusion strategy not only effectively preserves the time-frequency characteristics of the original signals in the image space but also enhances the representational capacity of the image features by leveraging the complementary information across the RGB channels, thereby providing highly discriminative input data for subsequent feature extraction and fault diagnosis using convolutional neural networks.

### 3.2. Design of the Improved ResNet-18 Network

The core principle of the attention mechanism in deep learning is to guide the model to focus on regions or features in the input that are most relevant to the current task, analogous to the human visual system’s ability to concentrate on key information while processing complex stimuli, thereby filtering out redundant data [28]. By dynamically allocating weights, the attention mechanism effectively enhances feature representation, improving both the discriminative power and predictive accuracy of the model.

**Figure 5:**
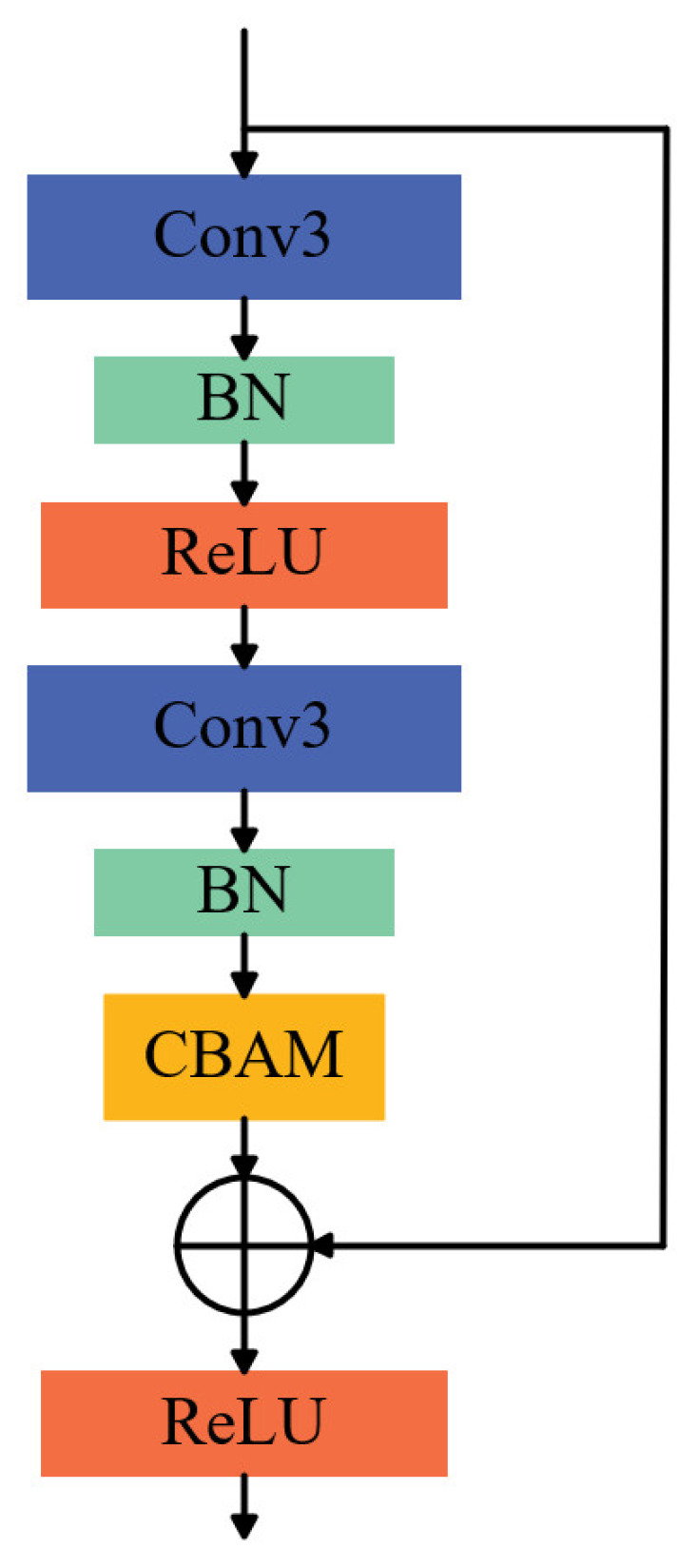
CBAM Residual Block.

**Figure 6:**
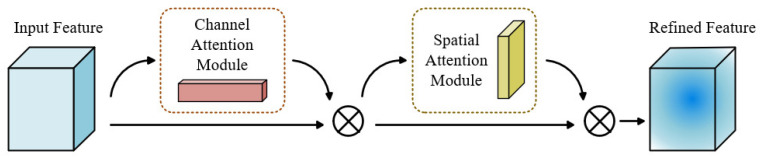
Structure of the CBAM.

**Figure 7:**
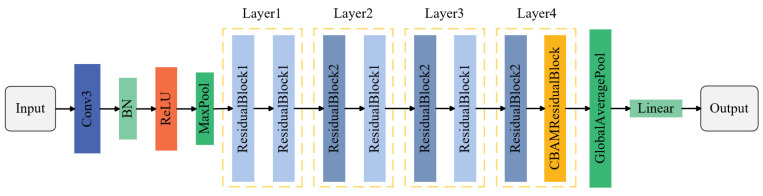
CBAM-ResNet-18 Network Architecture.

This study proposes a ResNet-18 network variant, CBAM-ResNet-18, which integrates channel and spatial attention mechanisms. Its core innovation lies in structurally modifying the original residual block to design a novel residual module (Figure 5). This module incorporates a Convolutional Block Attention Module (CBAM) [29] into the original residual block, replacing the conventional residual block with an enhanced version that integrates CBAM. This modification substantially improves the network’s capacity to represent input features and focus on critical information. The overall architecture of CBAM is illustrated in Figure 6, while the CBAM-ResNet-18 network structure is depicted in Figure 7. The algorithm describing the construction process of the CBAM-ResNet-18 network is presented in Algorithm 2.
**Algorithm 2.** Algorithmic description of the construction process of CBAM-ResNet-18 network**Step****Description****Input:**Input image size 224 × 224 × 3; kernel size *k*; stride *s*; padding p; number of filters *F_i_*; channel and spatial attention weights from CBAM.**Output:**Layer graph L_CBAM-ResNet18_ containing all convolutional, normalization, residual, and attention connections.**Step 1:**Initialize an empty layer graph L←∅.**Step 2:****Add input stem:** a. Add imageInputLayer with z-score normalization. b. Add a 7 × 7 convolution layer (64 filters, stride = 2, padding = 3). c. Apply batch normalization and ReLU activation. d. Add 3 × 3 max pooling (stride = 2).**Step 3:****Construct residual stages:** for each residual block group *G_i_*∈{*Res*2, *Res*3, *Res*4, *Res*5} do a. Main path (branch2): add two 3 × 3 convolution layers with batch normalization and ReLU. b. Shortcut path (branch1): if downsampling is required, add a 1 × 1 convolution (stride = 2). c. Merge: fuse the two branches via additionLayer(2) followed by ReLU activation. end for**Step 4:****Insert CBAM:** a. Detach the output of the final residual block (Res5b). b. Add CBAM subnetwork with two sequential attention units: —Channel attention: global average pooling → MLP → sigmoid weighting. —Spatial attention: concatenate max/avg feature maps → convolution → sigmoid. c. Reconnect CBAM output to the main residual path.**Step 5:****Add classification head:** a. Apply global average pooling. b. Add a fully connected layer (10 output neurons). c. Append softmax and classification layers.**Step 6:**Return the completed network graph *L_CBAM-ResNet18_***Notes:**The CBAM adaptively refines features via channel and spatial attention, improving discriminative capability while preserving ResNet-18’s computational efficiency.

**Figure 8:**
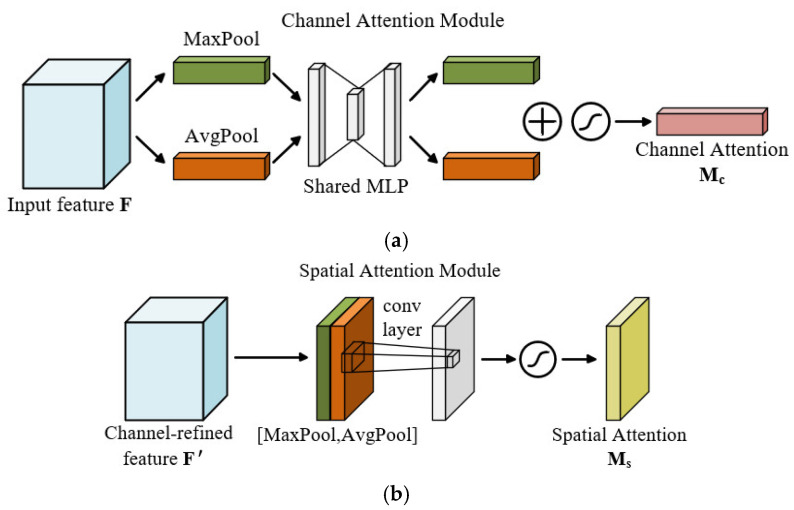
Structures of the CAM and SAM: (**a**) CAM; (**b**) SAM.

The CBAM comprises two sequential sub-modules: the Channel Attention Module (CAM) and the Spatial Attention Module (SAM) [30]. By adaptively learning both “which features to attend to” and “where to focus,” the module substantially enhances the network’s sensitivity to critical features, thereby improving overall feature extraction and enabling higher-precision fault diagnosis [31]. The detailed computational procedures of CAM and SAM are illustrated in Figure 8.

The CAM is designed to capture the global dependencies of feature maps along the channel dimension, determining “which channel features are most critical for the current task” [28]. Specifically, global average pooling and max pooling are applied along the spatial dimensions of the input feature map to generate two one-dimensional channel descriptors. These descriptors are then passed through a shared Multilayer Perceptron (MLP) for nonlinear transformation, summed, and finally processed through a sigmoid activation function to produce a channel attention weight map, which modulates the importance of each channel feature. The computation of CAM is expressed as follows:

(12) $$M_{c}\left(F)=\sigma (\mathrm{MLP}(\mathrm{AvgPool}(F))+\mathrm{MLP}(\mathrm{MaxPool}(F))\right)$$

Here, σ denotes the sigmoid function, while AvgPool(F) and MaxPool(F) represent average pooling and max pooling operations, respectively.

The SAM focuses on modeling the significance of spatial dimensions to guide the model in determining “which regions are more critical” [32]. First, Max pooling and average pooling are performed on the input feature map along the channel dimension to generate two single-channel two-dimensional feature maps. These feature maps are then concatenated along the channel axis and passed through a convolutional layer with a 7 × 7 kernel to integrate local spatial information. The resulting two-dimensional spatial attention map is activated via a sigmoid function and multiplied element-wise with the input feature map, thereby achieving spatially hierarchical attention weighting. The computation of SAM is as follows:

(13) $$M_{s}(F_{1})\;=\sigma (f([\mathrm{AvgPool}(F_{1}),\mathrm{MaxPool}(F_{1})]))$$

Here, $F_{1}$ denotes the input feature map, and *f* represents the 7 × 7 convolution operation.

### 3.3. Fault Diagnosis Procedure

**Figure 9:**
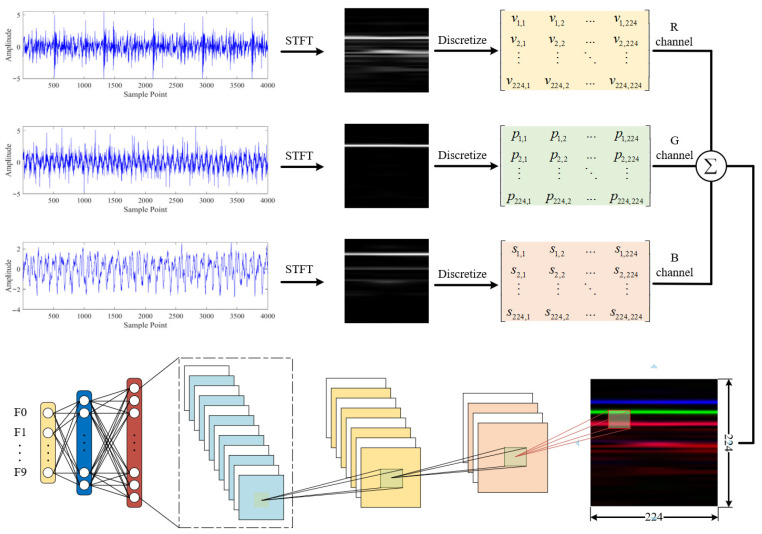
Fault Diagnosis Workflow.

To enable intelligent identification and classification of hydraulic pump health status, this study proposes a fault diagnosis method that integrates multi-source sensor information with a deep convolutional neural network. The approach fully leverages the complementary characteristics of multi-source signals, constructs a unified image representation through time-frequency information fusion, and exploits the powerful feature extraction capability of convolutional neural networks to perform fault diagnosis under complex operating conditions. The overall workflow is illustrated in Figure 9.

1. Data Collection: Operational data of the hydraulic pump under various operating conditions are acquired using sensors—such as vibration, pressure, and sound—placed at key locations on the pump.
2. Data Preprocessing: The raw signals are segmented into fixed-length intervals and normalized using Z-score standardization to make the amplitudes dimensionless, eliminating scale differences among different physical quantities.
3. RGB Image Construction: Each signal segment is transformed into a grayscale spectrogram using STFT and discretized into a grayscale matrix. The matrix amplitudes are then normalized to a 0–255 range. The grayscale matrices of the individual signals are assigned to the R, G, and B channels, respectively. Finally, the three matrices corresponding to the same time interval are combined to form a single RGB image.
4. Model Training: The resulting RGB images are fed into a convolutional neural network, with the dataset split into training and testing sets in appropriate proportions, to train the CNN model to recognize various fault types of the hydraulic pump.

## 4. Experimental Setup and Results Analysis

### 4.1. Dataset Description

**Figure 10:**
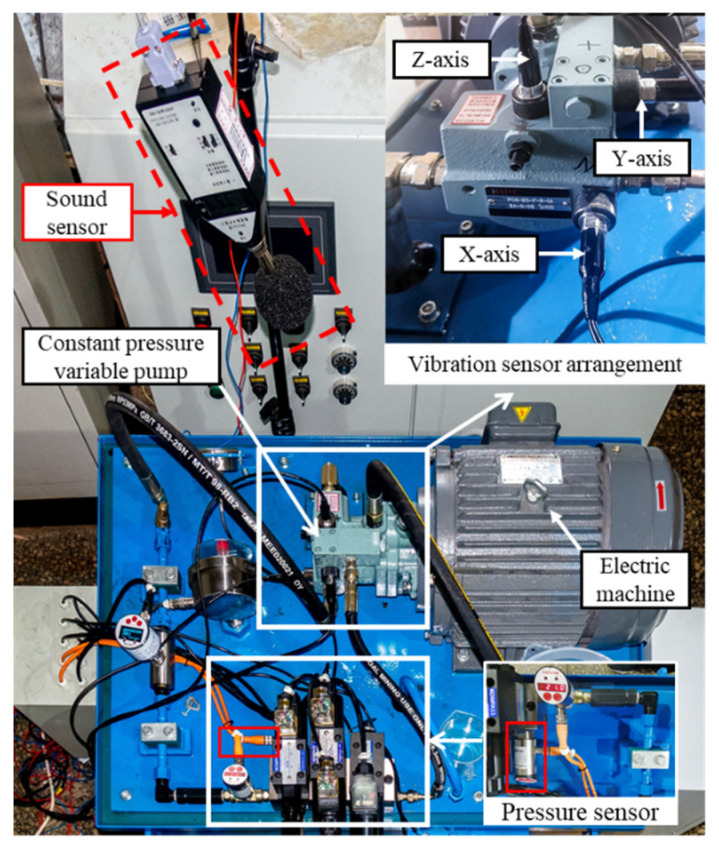
Sensor Installation Locations in the Constant-Pressure Variable Pump Fault Simulation Test System.

The constant-pressure variable pump fault simulation test bench is shown in Figure 10, with detailed information on the relevant components and their parameters provided in Table 2. The system is equipped with vibration sensors, pressure sensors, flow sensors, and sound level meters to monitor key physical quantities (such as vibration, pressure, and sound) during pump operation.

**Figure 11:**
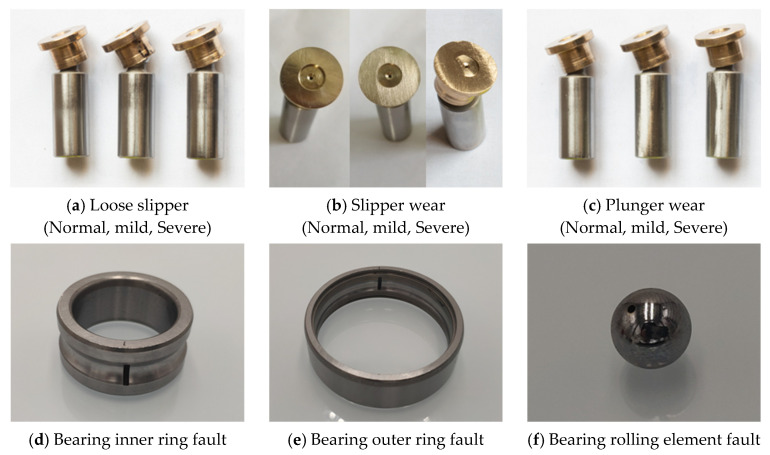
Physical images of fault components in the constant-pressure variable pump.

To ensure the authenticity and engineering relevance of the fault simulation, the experiment referred to typical fault types commonly observed under actual operating conditions of hydraulic pumps and employed fault injection technology to reconstruct the fault states (faulty components are shown in Figure 11). This method can accurately simulate the damage patterns of different components, enhancing the consistency between experimental data and actual fault data. A total of 10 operating states were established in this study, including one normal state and nine typical fault states (see Table 3), covering loose slipper faults (mild and severe), slipper wear (mild and severe), plunger wear (mild and severe), and bearing faults (inner ring, outer ring, and rolling elements). All experimental data were collected at a sampling frequency of 20 kHz, with each sample recorded for 5 s. The test environment was consistently set with an outlet temperature of 35–40 °C, an outlet pressure of 10 MPa, and a flow rate adjusted to 9 L/min.

### 4.2. Data Preprocessing and Hyperparameter Settings

The hydraulic pump fault simulation dataset used in this study encompasses 10 typical operating states. For each state, five segments of 5 s multi-source signals were collected, including vibration, pressure, and sound. Among the vibration signals, three directions (x, y, z) were measured, but only the x-direction vibration signal was selected in this study. To obtain a model with strong generalization capability and to ensure the reliability of experimental evaluation, the dataset was partitioned into a training set, validation set, and test set in a 60%:20%:20% ratio.

**Figure 12:**
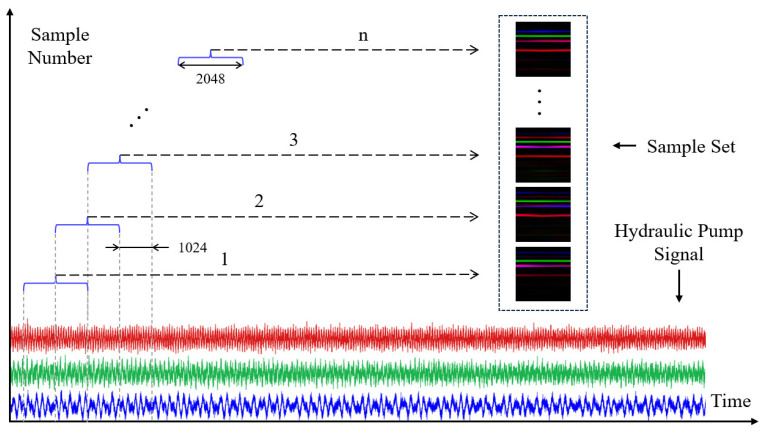
Construction process of RGB image samples.

Figure 12 illustrates the construction process of RGB image samples. The rated speed of the test bench motor is 1440 rpm, corresponding to approximately 834 sampling points per shaft rotation. To better capture potential fault features, the signals were segmented using a sliding window with a length of 2048 and a step size of 1024. This approach not only increases the number of samples but also mitigates the loss of features caused by signal truncation. Based on this, the three signals within each time window (X-direction vibration, pressure, and sound) are transformed into two-dimensional time-frequency images using STFT and sequentially mapped to the three channels of an RGB image: the red channel represents the vibration signal, the green channel represents the pressure signal, and the blue channel represents the sound signal, thereby fusing them into a single three-channel RGB image. Ultimately, by partitioning the dataset into training, validation, and test sets according to the specified proportions, a total of 2880 training samples, 960 validation samples, and 960 test samples were obtained, providing a dataset with good balance and representativeness in both scale and distribution.

**Figure 13:**
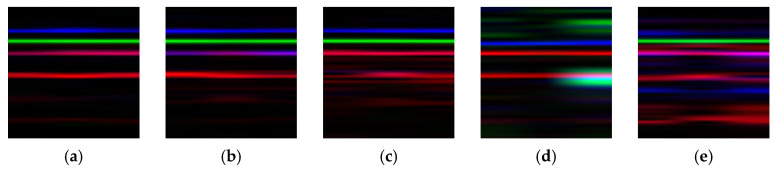

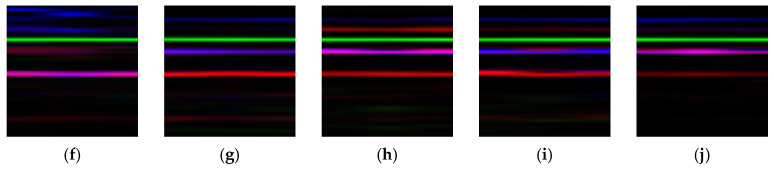
RGB images of the hydraulic pump under different fault conditions. (**a**) Normal; (**b**) Loose slipper (mild); (**c**) Loose slipper (severe); (**d**) Slipper wear (mild); (**e**) Slipper wear (severe); (**f**) Plunger wear (mild); (**g**) Plunger wear (severe); (**h**) Bearing inner ring fault; (**i**) Bearing outer ring fault; (**j**) Bearing rolling element fault.

Figure 13 presents RGB image samples of the hydraulic pump under various operating states. The figure shows clear differences in color distribution between normal operating conditions and various fault states. For instance, an increase in the energy of the vibration signal in the high-frequency band deepens the red regions, whereas variations in the pressure or sound signals result in noticeable changes in the green or blue regions. Such pronounced differences in color structure render the fault patterns highly separable in the image space.

During model training, a stochastic gradient descent optimizer is employed for parameter optimization, with an initial learning rate set to 0.01. A step learning rate schedule is applied, reducing the learning rate by a factor of 0.1 every two training epochs to enhance stability and convergence during the later stages of training. The training batch size is set to 128, and the maximum number of epochs is 10. During training, the model’s performance on the validation set is evaluated every 10 iterations to monitor its generalization capability.

The fault diagnosis framework proposed in this paper is implemented in the Matlab 2024a environment and is trained and tested on a local workstation equipped with an Intel Core i7-11700KF processor, a Gigabyte Z590 UD motherboard, and an NVIDIA GeForce RTX 2080 SUPER GPU.

### 4.3. Results Analysis

#### 4.3.1. Hydraulic Pump Fault Diagnosis Based on RGB-CBAM-ResNet-18

To comprehensively evaluate the performance of the proposed RGB-CBAM-ResNet-18 model for hydraulic pump fault diagnosis, this study conducts a systematic analysis from three perspectives: the training process, classification performance, and clustering effectiveness. First, the convergence speed and training stability of the model are assessed using accuracy and loss curves. Second, the confusion matrix is employed to visually illustrate the classification accuracy and misclassification for each fault category. Finally, by analyzing the clustering visualization of both input and output features, the model’s capability in feature extraction and class discrimination is further demonstrated.

**Figure 14:**
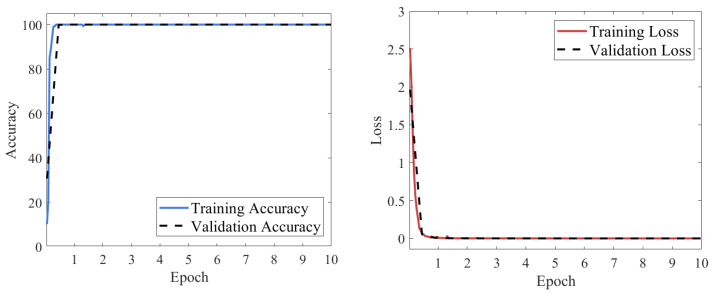
Loss and accuracy curves of the RGB-CBAM-ResNet-18 model.

Figure 14 presents the loss function and classification accuracy curves of the RGB-CBAM-ResNet-18 model during training. As shown, the model achieved a significant performance improvement within the first epoch, with the loss function rapidly decreasing to near zero and the accuracy quickly rising to almost 100%. From the second epoch onward, both the training and validation processes exhibited strong stability, with accuracy consistently maintained at a high level and loss values stabilizing at very low levels. No obvious overfitting or underfitting was observed throughout the training, indicating that the model possesses robust feature representation capability and excellent training stability in fusing multi-source features.

**Figure 15:**
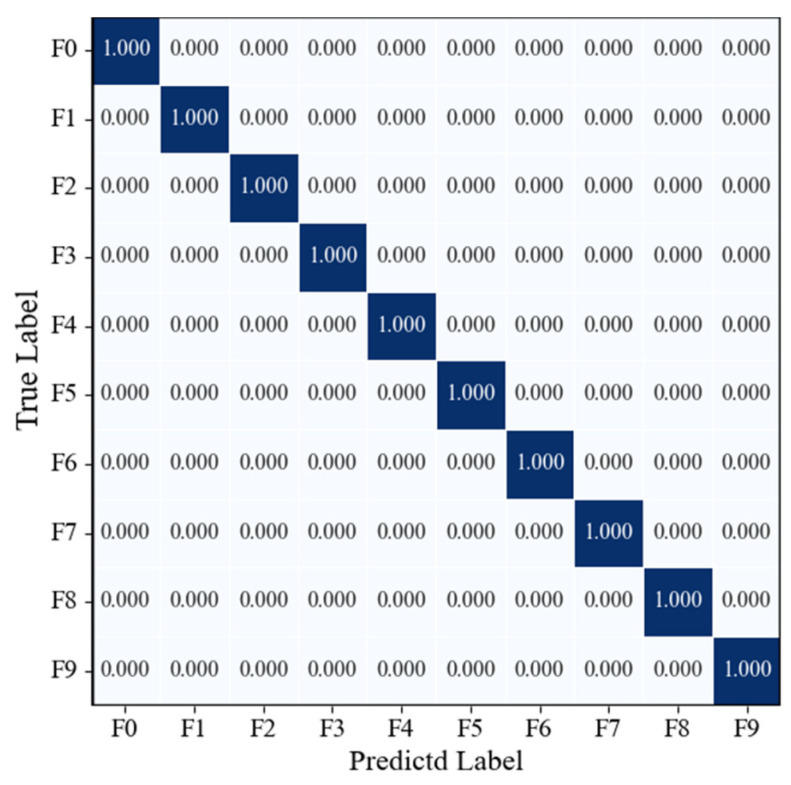
Confusion matrix of the RGB-CBAM-ResNet-18 model.

Figure 15 presents the confusion matrix on the model’s test set. It can be observed that all category samples were correctly classified, with the confusion matrix exhibiting a highly concentrated diagonal pattern and no misclassification or confusion between categories. The recall rate for each category reached 100%, fully demonstrating the model’s excellent capability in discriminating multi-source signal fusion features and its stable, reliable recognition performance across various operating conditions.

**Figure 16:**
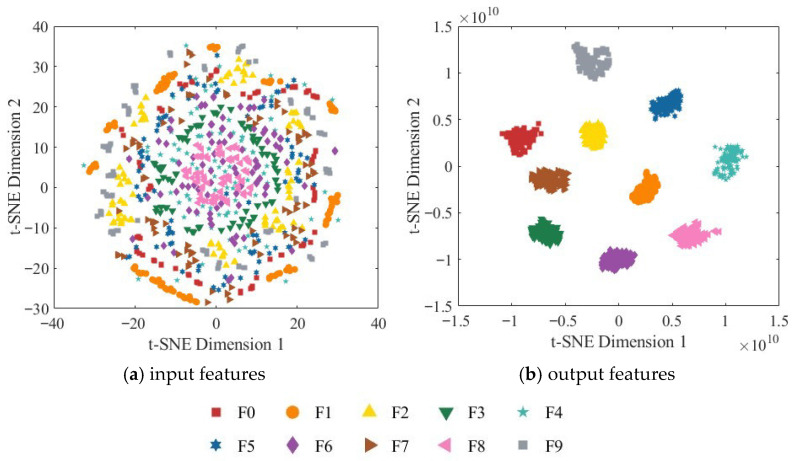
Two-dimensional t-SNE visualization of the RGB-CBAM-ResNet-18 model.

To further assess the model’s feature extraction capability, t-SNE (t-distributed Stochastic Neighbor Embedding) was employed to reduce the dimensionality of the node features and map them into a two-dimensional space for visualization. Figure 16 presents the two-dimensional t-SNE visualization of both the input and output features.

As shown in Figure 16a, the original input features are highly overlapping in the two-dimensional space, and the boundaries between different categories are indistinct, indicating that the input data is not separable in the original feature space. In contrast, the output features from the fully connected layer exhibit highly concentrated clusters with clear inter-class separation. Samples of different categories form distinct boundaries in the two-dimensional space and display significant intra-class cohesion, demonstrating that the model successfully extracts deep features with high discriminability. These results validate the effectiveness of the ResNet-18 network enhanced with the CBAM in strengthening spatial and channel attention and further demonstrate that the multi-source information fusion strategy can significantly improve the model’s classification performance.

#### 4.3.2. Comparative Analysis

To comprehensively evaluate the performance advantages of the proposed RGB-CBAM-ResNet-18 model and verify its effectiveness in hydraulic pump fault diagnosis, the traditional machine learning algorithm Support Vector Machine (SVM) was introduced as a baseline for comparative analysis. Owing to its strong generalization capability and nonlinear classification performance, SVM is commonly employed in small-sample learning scenarios for mechanical fault diagnosis, providing an important reference for assessing deep learning models [33,34,35]. To ensure consistency of input data, the baseline model first segments the vibration signals along the x-axis using a sliding window with a length of 2048 and a step size of 1024, yielding 480 segments per condition and a total of 4800 signal samples. Subsequently, the dataset is split into training and testing sets at a 70%:30% ratio, containing 3360 and 1440 samples, respectively.

The SVM model employed a radial basis function (RBF) kernel, with the penalty parameter C and kernel width γ set to 10.0 and 0.01, respectively. To ensure comparability with the deep learning model, ten features were manually extracted from the input data, including seven time-domain features (mean, standard deviation, maximum, minimum, root mean square, skewness, and kurtosis) and three frequency-domain features (spectral mean, spectral maximum, and spectral variance), forming a low-dimensional feature vector as the SVM input. The feature vectors were then normalized to the [0,1] range to eliminate the influence of differing scales on classification. This baseline experiment effectively assesses the diagnostic performance of conventional shallow classifiers under identical data conditions, providing a quantitative reference for evaluating the improvements achieved by the subsequent deep learning model.

Building on this, systematic comparative experiments were further designed at both the input feature construction and network architecture levels. The baseline models, CWT-ResNet-18 and STFT-ResNet-18, transform the single-channel vibration signals along the x-axis into two-dimensional time–frequency images using the Continuous Wavelet Transform (CWT) and STFT, respectively. Meanwhile, the baseline models are trained using the original, unmodified ResNet-18 network to isolate the individual effects of the fusion strategy and network architecture improvements. To ensure an objective comparison, the experiments use identical vibration signals, training parameters, and evaluation metrics, allowing a horizontal assessment of model diagnostic performance. Additionally, the baseline models segment the signals using a sliding window with a length of 2048 and a step size of 1024, and the dataset is partitioned into training, validation, and testing sets in a 60%:20%:20% ratio, yielding 2880 training samples, 960 validation samples, and 960 testing samples.

**Figure 17:**
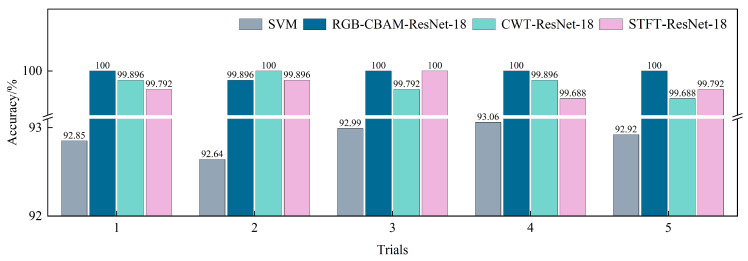
Comparison of classification accuracy over five independent repeated experiments.

To comprehensively evaluate the stability and generalization capability of the proposed method, five independent repeated experiments were conducted for the RGB-CBAM-ResNet-18 model, the SVM model, the CWT-ResNet-18 model, and the STFT-ResNet-18 model. The results show that the average classification accuracies were 99.97% for RGB-CBAM-ResNet-18, 92.89% for SVM, 99.85% for CWT-ResNet-18, and 99.83% for STFT-ResNet-18. Figure 17 presents the comparison of classification accuracies across the five trials for the four models. The results indicate that the improved RGB-CBAM-ResNet-18 consistently achieves higher recognition accuracy with lower performance variability, significantly outperforming SVM, CWT-ResNet-18, and STFT-ResNet-18, thereby further validating the effectiveness and robustness of the proposed approach in hydraulic pump fault diagnosis.

**Figure 18:**
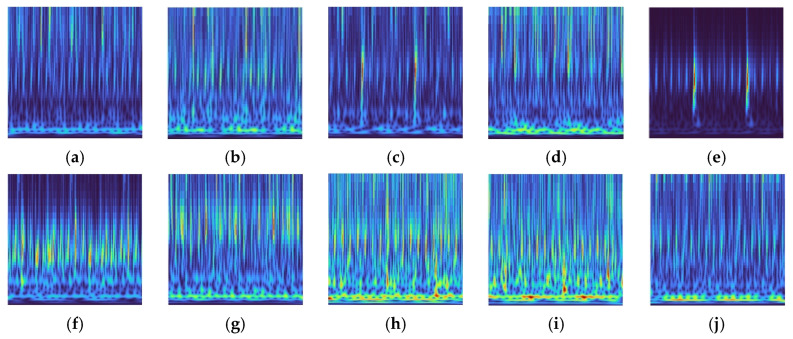
CWT image samples of the hydraulic pump under different fault states. (**a**) Normal; (**b**) Loose slipper (mild); (**c**) Loose slipper (severe); (**d**) Slipper wear (mild); (**e**) Slipper wear (severe); (**f**) Plunger wear (mild); (**g**) Plunger wear (severe); (**h**) Bearing inner ring fault; (**i**) Bearing outer ring fault; (**j**) Bearing rolling element fault.

Figure 18 presents the CWT image samples of the hydraulic pump under different operating states. Most of the images exhibit longitudinal texture patterns, yet clear differences remain between categories. Under normal operation, the energy distribution is relatively uniform with no noticeable abnormal textures. Loose slipper and slipper wear are primarily characterized by enhanced high-frequency textures, and in cases of severe slipper wear, the low-frequency energy bands are markedly weakened or even absent. Plunger wear leads to abnormal energy distribution in the mid- and low-frequency bands, whereas bearing faults present local high-energy pulses while retaining periodic textures. These distinctive time-frequency features provide a reliable basis for feature extraction and accurate classification in subsequent network processing.

**Figure 19:**
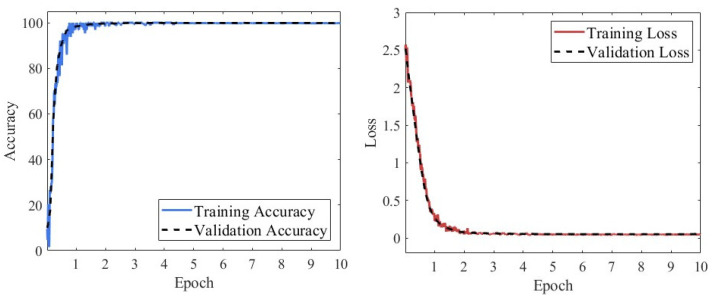
Loss and accuracy curves of the CWT-ResNet-18 model during training and validation.

Figure 19 presents the loss and accuracy curves of the CWT-ResNet-18 model during training and validation. The model demonstrated a certain degree of convergence during training: the training accuracy increased rapidly in the first three epochs, rising from near 0% to approximately 95%, then continued to increase slowly and reached saturation after the fifth epoch, eventually stabilizing above 99%. The loss also decreased sharply to below 0.1 within the first three epochs and then gradually approached zero. Although the overall accuracy was high, fluctuations in the early training curves indicate some instability in the model’s convergence process. This phenomenon may be attributed to the fact that the CWT-ResNet-18 model constructs feature maps solely from X-direction vibration signals, limiting its ability to perceive complex operating condition features and effectively capture multi-dimensional discriminative information, thereby affecting the overall diagnostic performance.

**Figure 20:**
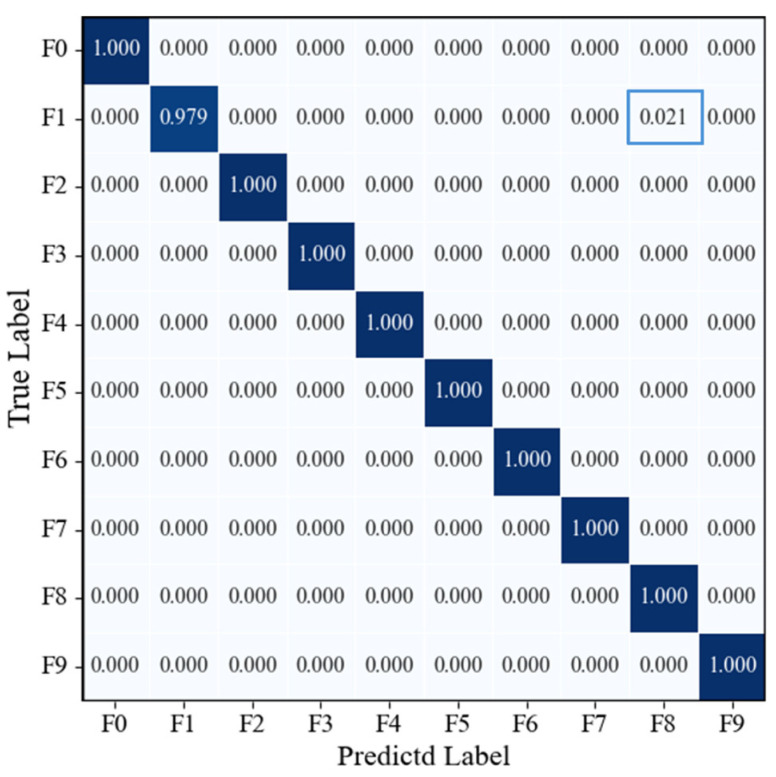
Confusion matrix of the CWT-ResNet-18 model on the test set.

Figure 20 presents the confusion matrix of the CWT-ResNet-18 model on the test set. It can be observed that, although the recall rates for most categories are high, indicating that the model possesses a certain overall classification capability, some category confusion remains. For instance, a few misclassifications occur between Class 1 and Class 8, suggesting that the model’s ability to differentiate specific features of certain operating condition signals is still limited. Due to the relatively single source of input information, the model’s overall recognition capability is constrained. Overall, the CWT-ResNet-18 model demonstrates a moderate level of classification performance, but its discriminative ability between categories is slightly inferior to that of the RGB-CBAM-ResNet-18 model.

**Figure 21:**
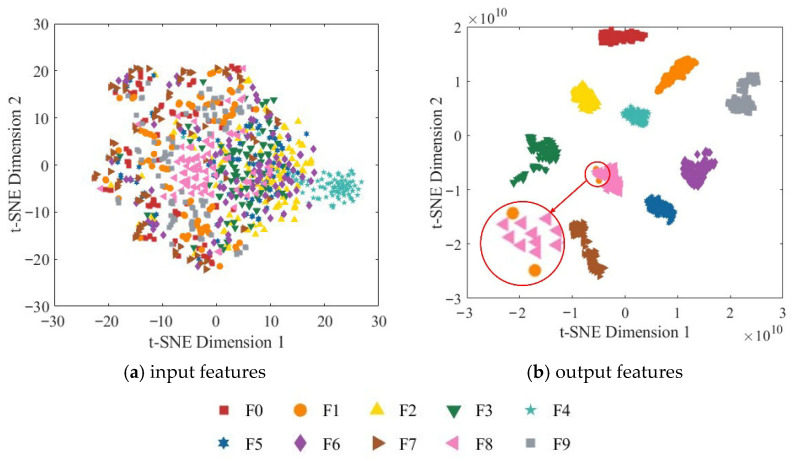
Two-dimensional t-SNE visualization of input and output features for the CWT-ResNet-18 model.

Figure 21 illustrates the distribution of input and output features of the CWT-ResNet-18 model in the t-SNE space. The input features exhibit a highly scattered distribution with indistinct boundaries between categories. In contrast, the output features from the fully connected layer show a markedly improved distribution, with most categories forming relatively well-defined clusters, indicating that the model possesses a certain feature extraction capability. However, some category confusion remains; for instance, a small number of Class 1 samples are mixed within Class 8, suggesting that the model still exhibits some ambiguity in distinguishing working condition categories with similar boundaries.

**Figure 22:**
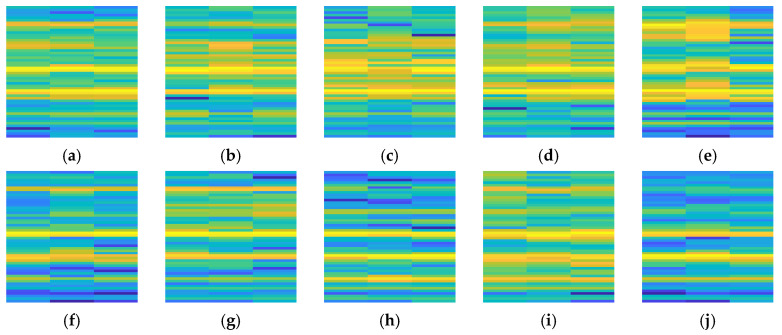
STFT image samples of the hydraulic pump under different fault conditions. (**a**) Normal; (**b**) Loose slipper (mild); (**c**) Loose slipper (severe); (**d**) Slipper wear (mild); (**e**) Slipper wear (severe); (**f**) Plunger wear (mild); (**g**) Plunger wear (severe); (**h**) Bearing inner ring fault; (**i**) Bearing outer ring fault; (**j**) Bearing rolling element fault.

Figure 22 presents the STFT image samples of the hydraulic pump under different operating states. Overall, most images exhibit a transverse stripe pattern dominated by mid- to low-frequency components. In the normal state, the stripes are evenly distributed with no obvious anomalies. Wear of the loose slipper and slipper wear is characterized by pronounced stripes in the mid- to high-frequency regions, while plunger wear primarily manifests as uneven energy distribution in the mid- to low-frequency bands. Bearing faults are accompanied by local energy anomalies superimposed on the overall stripe pattern. Although the inter-class separability of STFT images is relatively limited, differences in frequency band energy distribution and local texture still provide valuable cues for feature extraction and classification in subsequent network processing.

**Figure 23:**
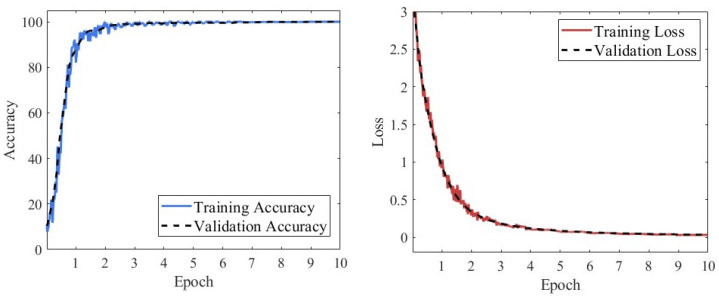
Loss and accuracy curves of the STFT-ResNet-18 model during training and validation.

Figure 23 illustrates the loss and accuracy trends of the STFT-ResNet-18 model during training and validation, with the curves remaining relatively stable overall. Training accuracy rapidly increased to over 90% within the first two epochs, then gradually rose and stabilized around the seventh epoch, ultimately exceeding 99%. Meanwhile, the training loss decreased from an initially high level to below 0.2 during the first three epochs, then continued to decline slowly, gradually converging toward near-zero in the later stages. Although the overall convergence was satisfactory, some fluctuations were still observed throughout the training process.

**Figure 24:**
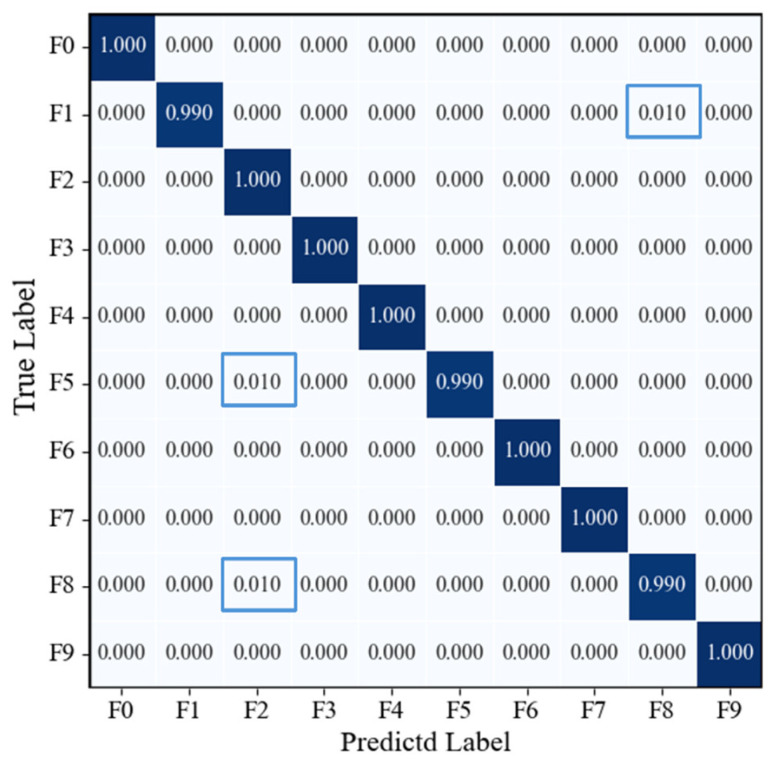
Confusion matrix of the STFT-ResNet-18 model on the test set.

Figure 24 presents the confusion matrix of the STFT-ResNet-18 model on the test set. The results indicate that STFT-ResNet-18 achieves high classification accuracy for most categories, demonstrating strong feature extraction and recognition capabilities. However, some category confusion persists, such as minor misclassifications between Class 1 and Class 8, Class 5 and Class 2, and Class 8 and Class 2. Although the overall performance of the model is robust, certain limitations remain in distinguishing specific categories.

**Figure 25:**
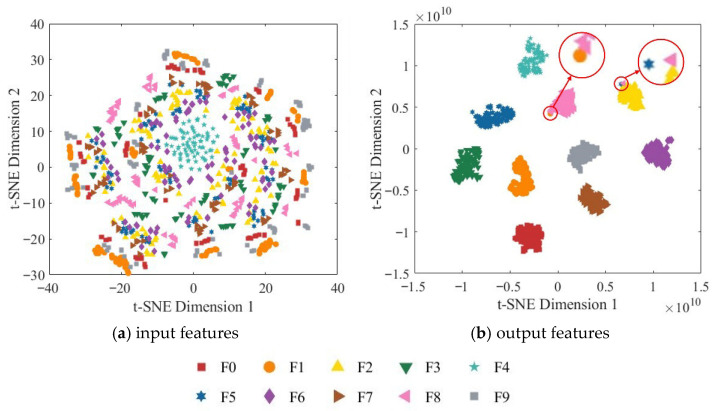
Two-dimensional t-SNE visualization of input and fully connected layer output features of the STFT-ResNet-18 model.

Figure 25 illustrates the distribution of the STFT-ResNet-18 model’s input features and the fully connected layer’s output features in the t-SNE space. The input features are chaotically distributed, with unclear boundaries between categories and no distinct clustering structure. In contrast, the output features from the fully connected layer are markedly more clustered, showing significantly improved inter-class separability, with most categories forming relatively compact clusters. However, some category confusion remains; for instance, a few Class 1 samples are mixed into Class 8, and minor overlaps are observed between Class 2 and Class 5 as well as Class 2 and Class 8, indicating that the discriminative boundaries for certain categories are still not fully distinct.

To further evaluate the impact of the improved model on computational resource consumption, four key metrics were selected for quantitative analysis: Model File Size, number of parameters (Params), inference latency (Latency), and millions of floating-point operations (MFLOPs). Params were obtained by counting all learnable parameters in the network and converting them to millions to measure the complexity of the model structure. Model File Size represents the actual storage space occupied by the trained network model, reflecting its storage requirements for edge deployment. Latency was measured by repeatedly performing forward classification on a single sample from the test set and averaging the time taken, indicating the model’s response speed and real-time processing capability during inference. MFLOPs denotes the number of floating-point operations required during inference, serving as a core indicator of computational load and reflecting the model’s dependence on computing resources.

As shown in Table 4, the RGB-CBAM-ResNet-18 model achieves a classification accuracy of 99.97%, surpassing CWT-ResNet-18 (99.85%), STFT-ResNet-18 (99.83%), and the baseline SVM model (92.89%), indicating superior feature discriminative capability. In terms of computational resources, the three deep convolutional models have parameter counts (Params) of 11.27 M, 11.18 M, and 11.13 M, with model file sizes of 39.66 MB, 39.33 MB, and 39.27 MB, respectively, showing minimal differences, while the SVM model has only 0.3 M Params and a 0.24 MB model file size. Regarding floating-point operations (MFLOPs), RGB-CBAM-ResNet-18 requires 2494.64 MFLOPs, slightly higher than CWT-ResNet-18 and STFT-ResNet-18, whereas SVM requires only 0.14 MFLOPs. The inference latency of RGB-CBAM-ResNet-18 is 8.27 ms, slightly higher than CWT-ResNet-18 (5.70 ms) and STFT-ResNet-18 (5.62 ms), while SVM exhibits a latency of only 0.07 ms. Overall, RGB-CBAM-ResNet-18 significantly improves classification performance; although its computational overhead is several times higher than that of SVM, it remains within an acceptable range. For lightweight applications, SVM still offers advantages in rapid inference and low resource consumption. This comparison demonstrates that the proposed model achieves a favorable balance between classification accuracy and computational complexity.

In summary, compared with control models that rely solely on a single vibration signal, the proposed model enhances the representational capacity of input features through RGB fusion of multi-source signals. Furthermore, the integration of the CBAM further optimizes the network’s feature extraction capability, resulting in significant improvements in both accuracy and stability.

## 5. Conclusions

This paper addresses the key challenges in hydraulic pump fault diagnosis by proposing an intelligent fault diagnosis method that leverages multi-source signal fusion combined with a dual attention mechanism. The method transforms vibration, pressure, and sound signals into fused RGB image representations and incorporates channel and spatial attention mechanisms to enhance the network’s feature representation and discriminative capability, thereby enabling high-precision identification and classification of multiple fault states in hydraulic pumps. The main conclusions are summarized as follows:

(1) In this paper, time-frequency features of vibration, pressure, and sound signals are extracted to construct uniform-scale RGB images, enabling visualization of multi-source data fusion and adapting to the input requirements of convolutional neural networks. Experimental results demonstrate that this fusion strategy significantly improves fault identification performance compared with single-signal input.
(2) Based on the ResNet-18 architecture, an improved approach incorporating a dual attention mechanism was proposed, and a novel residual module integrating both channel and spatial attention mechanisms was designed. Comparative experiments verified the network’s superior capability in feature extraction and classification performance.
(3) An intelligent fault diagnosis framework integrating multi-source signal fusion with the improved residual neural network was developed to achieve automatic identification and classification of multiple hydraulic pump fault states, enhancing the intelligence of the diagnostic process and demonstrating high diagnostic accuracy and strong engineering applicability.

In the fault simulation experiments of this study, vibration signals in the x, y, and z directions of the hydraulic pump were collected; however, only the x-direction signal was used to construct the dataset, and the diagnostic effectiveness of signals from different directions was not systematically evaluated. Future research could employ principal component analysis to quantitatively assess the information contribution of the three-axis signals, allowing for the selection of the most representative vibration signal and further improving the overall performance of the fault diagnosis model.

## Figures and Tables

**Table 1:** Major Advantages and Disadvantages of Data Fusion Methods.

Fusion Level	Advantages	Disadvantages
Data-level Fusion	Retains the most complete raw information, providing richer data support for end-to-end feature learning in deep learning models.	Requires strict time synchronization and signal normalization; sensitive to noise; high-dimensional data may increase computational and storage costs.
Feature-level Fusion	Balances information retention with model adaptability, enhancing the discriminative capability of feature representations.	Relies on manual feature extraction and selection; low degree of automation in the modeling process; sensitive to feature weighting and fusion strategies.
Decision-level Fusion	Exhibits good robustness and scalability; suitable for multi-model ensembles and cross-domain diagnostic scenarios.	Only fuses final outputs, failing to effectively utilize raw data and intermediate feature information, which may reduce diagnostic accuracy.

**Table 2:** Key Parameters of Major Components of the Test Bench.

Component	Model	Specifications
Constant-Pressure Variable Pump	P08-B3-F-R-01	Displacement: 8 mL/r
		Rated Pressure: 3~21 MPa
		Rated Speed: 500~2000 r/min
Drive Motor	C07-43BO	Rated Power: 5.5 kW
		Rated Speed: 1440 r/min
Accelerometer	PCB M603C01	Sensitivity: 100 mV/g
		Range: ±50 g
		Frequency Response: 0.5 Hz~10 kHz
Pressure Sensor	PS300-B250G1/2MA3P	Measurement Range: 0~25 MPa
		Supply Voltage: 12~30 VDC
		Output Signal: 4~20 mA
Sound Level Meter	AWA5661	Measurement Range: 25~140 dB
		Sensitivity: 40 mV/Pa
		Frequency Range: 10 Hz~16 kHz

**Table 3:** Fault Types and Settings of the Constant-Pressure Variable Pump.

No.	Fault Type	Setting Method	Label
1	Normal	—	F0
2	Loose Slipper (mild)	Use a plunger slipper assembly with a certain degree of looseness; slipper clearance: 0.24 mm	F1
3	Loose Slipper (Severe)	Use a plunger slipper assembly with a certain degree of looseness; slipper clearance: 0.48 mm	F2
4	Slipper Wear (mild)	Grind the slipper with 80-grit sandpaper until its mass decreases by 0.2 g and slightly uneven wear is applied	F3
5	Slipper Wear (Severe)	Grind the slipper with 40-grit sandpaper until its mass decreases by 0.6 g and slightly uneven wear is applied	F4
6	Plunger Wear (mild)	Grind the plunger with 180-grit sandpaper until its mass decreases by 0.15 g	F5
7	Plunger Wear (Severe)	Grind the plunger with 100-grit sandpaper until its mass decreases by 0.45 g	F6
8	Bearing Inner Ring Fault	Use EDM to machine a 1 mm wide × 1 mm deep groove through the inner ring raceway along the perpendicular direction	F7
9	Bearing Outer Ring Fault	Use EDM to machine a 1 mm wide × 1 mm deep groove through the outer ring raceway along the perpendicular direction	F8
10	Bearing Rolling Element Fault	Use EDM to machine a 1 mm diameter × 1 mm deep pit on a rolling element of the bearing	F9

**Table 4:** Comparison of model classification performance and computational resource consumption.

Model	Acc	Model File Size (M)	Params (M)	MFLOPs	Latency (ms)
RGB-CBAM-ResNet-18	99.97	39.66	11.27	2494.64	8.27
SVM	92.89	0.24	0.03	0.14	0.07
CWT-ResNet-18	99.85	39.33	11.18	1733.89	5.70
STFT-ResNet-18	99.83	39.27	11.13	1727.33	5.62

## Data Availability

The data presented in this study are available on request from the corresponding author.

## References

[1] Zhu Y., Li G., Tang S., Wang R., Su H., Wang C. (2022). Acoustic signal-based fault detection of hydraulic piston pump using a particle swarm optimization enhancement CNN. Appl. Acoust..

[2] Xia S., Huang W., Zhang J. (2025). A novel fault diagnosis method based on nonlinear-CWT and improved YOLOv8 for axial piston pump using output pressure signal. Adv. Eng. Inform..

[3] Liu S., Yin J., Zhang Y., Liang P. (2025). MLIFT: Multi-scale linear interaction fusion transformer for fault diagnosis of hydraulic pumps. Measurement.

[4] Wen L., Li X., Gao L., Zhang Y. (2018). A new convolutional neural network-based data-driven fault diagnosis method. IEEE Trans. Ind. Electron..

[5] Zhu Y., Li G., Wang R., Tang S., Su H., Cao K. (2021). Intelligent fault diagnosis of hydraulic piston pump combining improved LeNet-5 and PSO hyperparameter optimization. Appl. Acoust..

[6] Xu Z., Li C., Yang Y. (2021). Fault diagnosis of rolling bearings using an improved multi-scale convolutional neural network with feature attention mechanism. ISA Trans..

[7] Xiao Q., Li S., Zhou L., Shi W. (2022). Improved variational mode decomposition and CNN for intelligent rotating machinery fault diagnosis. Entropy.

[8] Wang S., Xiang J., Zhong Y., Tang H. (2018). A data indicator-based deep belief networks to detect multiple faults in axial piston pumps. Mech. Syst. Signal Process..

[9] Zhao R., Yan R., Chen Z., Mao K., Wang P., Gao R.X. (2019). Deep learning and its applications to machine health monitoring. Mech. Syst. Signal Process..

[10] Eang C., Lee S. (2025). Predictive maintenance and fault detection for motor drive control systems in industrial robots using CNN-RNN-based observers. Sensors.

[11] Lian C., Zhao Y., Sun T., Dong F., Zhan Z., Xin L. (2024). A new time series data imaging scheme for mechanical fault diagnosis. IEEE Trans. Instrum. Meas..

[12] Yu X., Wang Y., Liang Z., Shao H., Yu K., Yu W. (2023). An adaptive domain adaptation method for rolling bearings’ fault diagnosis fusing deep convolution and self-attention networks. IEEE Trans. Instrum. Meas..

[13] Kumar A., Gandhi C.P., Zhou Y., Kumar R., Xiang J. (2020). Improved deep convolution neural network (CNN) for the identification of defects in the centrifugal pump using acoustic images. Appl. Acoust..

[14] Xu Y., Li Z., Wang S., Li W., Sarkodie-Gyan T., Feng S. (2021). A hybrid deep-learning model for fault diagnosis of rolling bearings. Measurement.

[15] Wang H., Xu J., Yan R., Gao R.X. (2020). A new intelligent bearing fault diagnosis method using SDP representation and SE-CNN. IEEE Trans. Instrum. Meas..

[16] Wang H., Li S., Song L., Cui L. (2019). A novel convolutional neural network based fault recognition method via image fusion of multi-vibration-signals. Comput. Ind..

[17] Shao H., Lin J., Zhang L., Galar D., Kumar U. (2021). A novel approach of multisensory fusion to collaborative fault diagnosis in maintenance. Inf. Fusion.

[18] Zhang X., Zhang X., Liu J., Wu B., Hu Y. (2023). Graph features dynamic fusion learning driven by multi-head attention for large rotating machinery fault diagnosis with multi-sensor data. Eng. Appl. Artif. Intell..

[19] Xia M., Li T., Xu L., Liu L., de Silva C.W. (2018). Fault diagnosis for rotating machinery using multiple sensors and convolutional neural networks. IEEE/ASME Trans. Mechatron..

[20] Zhong S., Fu S., Lin L. (2019). A novel gas turbine fault diagnosis method based on transfer learning with CNN. Measurement.

[21] Yang B., Lei Y., Jia F., Xing S. (2019). An intelligent fault diagnosis approach based on transfer learning from laboratory bearings to locomotive bearings. Mech. Syst. Signal Process..

[22] Vashishtha G., Chauhan S., Sehri M., Hebda-Sobkowicz J., Zimroz R., Dumond P., Kumar R. (2024). Advancing machine fault diagnosis: A detailed examination of convolutional neural networks. Meas. Sci. Technol..

[23] Zhang W., Peng G., Li C., Chen Y., Zhang Z. (2017). A new deep learning model for fault diagnosis with good anti-noise and domain adaptation ability on raw vibration signals. Sensors.

[24] Huang B., Liu J., Zhang Q., Liu K., Li K., Liao X. (2022). Identification and classification of aluminum scrap grades based on the Resnet18 model. Appl. Sci..

[25] Zhang Y., Xing K., Bai R., Sun D., Meng Z. (2020). An enhanced convolutional neural network for bearing fault diagnosis based on time–frequency image. Measurement.

[26] Zhu Z., Peng G., Chen Y., Gao H. (2019). A convolutional neural network based on a capsule network with strong generalization for bearing fault diagnosis. Neurocomputing.

[27] Kibrete F., Engida Woldemichael D., Shimels Gebremedhen H. (2024). Multi-sensor data fusion in intelligent fault diagnosis of rotating machines: A comprehensive review. Measurement.

[28] Guo J., Yang Y., Li H., Dai L., Huang B. (2024). A parallel deep neural network for intelligent fault diagnosis of drilling pumps. Eng. Appl. Artif. Intell..

[29] Woo S., Park J., Lee J.Y., Kweon I.S. (2018). CBAM: Convolutional block attention module. Lecture Notes in Computer Science.

[30] Jiang K., Xie T., Yan R., Wen X., Li D., Jiang H., Jiang N., Feng L., Duan X., Wang J. (2022). An Attention Mechanism-Improved YOLOv7 Object Detection Algorithm for Hemp Duck Count Estimation. Agriculture.

[31] Yan S., Wei H., Yang C., Liu Z., Zhang S., Zhao L. Fault diagnosis of rolling bearing based on CBAM-ICNN. Proceedings of the 2024 39th Youth Academic Annual Conference of Chinese Association of Automation (YAC).

[32] He C., Yasenjiang J., Lv L., Xu L., Lan Z. (2024). Gearbox fault diagnosis based on MSCNN-LSTM-CBAM-SE. Sensors.

[33] Chen J., Liu Q., Gao L. (2021). Deep convolutional neural networks for tea tree pest recognition and diagnosis. Symmetry.

[34] Jing L., Wang T., Zhao M., Wang P. (2017). An adaptive multi-sensor data fusion method based on deep convolutional neural networks for fault diagnosis of planetary gearbox. Sensors.

[35] Zhang C.-L., Zhang B.-X., Xu J.-H., Chen Z.-L., Zheng X.-Y., Zhu K.-Q., Xie H., Bo Z., Yang Y.-R., Wang X.-D. (2025). Fault diagnosis and removal for hybrid power generation systems based on an ensemble deep learning diagnostic method with self-healing strategies. Int. J. Hydrogen Energy.

